# 

**Published:** 1887-03

**Authors:** 


### Bristol Medico-Chirurgical Journal.

### MARCH, 1887.

### CONTENTS.

Original Papers:— *Page.*

Ought Craniotomy to be Abolished? By Joseph Griffiths Swayne, M.D. ... 1

On Graves's Disease, with a Case. By J. Michell Clarke, M.A., M.B. Cantab. ... 17

Clinical Records:—

Ill Effects of a Small Aneurism of the Aortic Arch Illustrated. By Henry F. A. Goodridge, M.D., F.R.C.P. ... 30

A Case of Suppurating Middle Ear, Thrombosis and Phlebitis of Lateral Sinus, Death. By W. H. Harsant, F.R.C.S. ... 37

Periscope ... 43

Notes on Preparations for the Sick ... 58

Reviews of Books ... 64

Scraps ... 71

